# Timely care for age-related macular degeneration: a qualitative study among retina specialists in Israel

**DOI:** 10.1186/s13584-024-00616-w

**Published:** 2024-06-04

**Authors:** Vicki Myers, Osnat Luxenburg, Rachel Wilf-Miron, Hani Levkovitch Verbin

**Affiliations:** 1 Center for Healthcare Technology and Innovation Policy Research, Gertner Institute of Epidemiology & Health Policy Research, Sheba Medical Center, Ramat Gan, Israel; 2 Medical Technology, Health Information and Research Directorate, Ministry of Health, Jerusalem, Israel; 3 School of Public Health, Faculty of Medical & Health Sciences, Tel Aviv University, Ramat Aviv, Israel; 4 Ophthalmology Department, Faculty of Medical & Health Sciences, Tel Aviv University, Ramat Aviv, Israel; 5 Glaucoma Service, Goldschleger Eye Institute, Sheba Medical Center, Tel-Hashomer, Israel

**Keywords:** Age-related macular degeneration (AMD), Retina, Qualitative research, Health services

## Abstract

**Background:**

Age-related macular degeneration (AMD) affects quality of life and independence, and its incidence and prevalence are increasing due to ageing of the population. Access to effective timely treatment can improve vision and reduce incidence of blindness. This study aimed to explore the perspectives of ophthalmologists in the Israeli public healthcare system regarding timely treatment of AMD patients.

**Methods:**

Qualitative semi-structured interviews were conducted in 2020-2021 with 22 senior ophthalmologists, from 10 general hospitals and from two HMOs, representing different geographic regions. All interviewees specialize in retinal diseases and work with AMD patients. Interviews discussed patient pathways involved in the diagnosis and treatment of AMD, access to care, and obstacles to timely care. Thematic analysis was conducted.

**Results:**

Based on the interviews, we describe the usual referral and treatment pathways. Themes included regional disparities, long wait times in some areas, a lack of retina specialists, differences in referral pathways, inappropriate use of emergency department to obtain timely treatment, and second-line treatment not fully covered by insurance, most affecting the weakest segments of the population.

**Conclusions:**

Loss of vision incurs high health and societal costs. In the context of insufficient medical manpower in Israel, the healthcare system will need to assess future resources to cope with accumulating burden of AMD cases over time in an ageing population. Precise referral information, and simultaneous referral to imaging and retinal clinics, may minimize delays in treatment. Awareness of AMD symptoms and the importance of early intervention could be highlighted by campaigns, particularly among high-risk groups.

**Highlights:**

• Interviews with hospital-based and community ophthalmologists showed regional disparities in AMD treatment, with long wait times and a lack of retina specialists in some areas.

• Differences in referral pathways, inappropriate use of emergency department to obtain timely treatment, and second line treatment not fully covered by insurance were highlighted.

• The healthcare system will need to assess future resources to cope with accumulating burden of AMD cases over time in an ageing population

• Precise referral information, and simultaneous referral to imaging and retinal clinics, may minimize delays in treatment.

• Awareness of AMD symptoms and the importance of early intervention should be emphasized in high-risk groups.

## Introduction

Age-related macular degeneration (AMD) is responsible for around 50% of cases of legal blindness around the world [1]. Patients with AMD suffer a significant decline in quality of life and increased dependence [2]. According to a systematic review conducted in 2014, AMD is projected to afflict 288 million people around the world by 2040, with a current pooled prevalence of 8% at age range 45-85 [3]. Early stage AMD is more prevalent than late stage, or neovascular (‘wet’) AMD (8% and 0.4%, respectively) [4]. Meta-analysis of the literature published in both English and Chinese databases up to March 2021, estimated the projected number of new cases of early and late AMD in 2050 would be 39.05 million and 6.41 million, respectively [5].

In the last decade, intravitreal anti-vascular endothelial growth factor (anti-VEGF) or anti-angiogenic therapy has become the standard of care in neovascular AMD, demonstrating efficacy in preventing vision loss [6]. The three main drugs used are Bevacizumab (Avastin), Ranibizumab (Lucentis) and Ilea.

Timeliness of treatment is crucial in AMD. Access to effective and timely treatment can improve vision and reduces the incidence of blindness [7]. Delays can lead to deterioration of sight and irreversible damage, with patients benefitting less from treatment. Best practice guidelines developed by a UK group of retinal specialists recommend early diagnosis, referral to a macular clinic within 1-2 days, and prompt access to treatment within 14 days of initial referral [6]. Improvement following treatment is often assessed by gain in letters in the visual acuity chart and OCT (ocular coherence tomography) parameters [8].

AMD affects mostly older populations, who are frailer, more vulnerable, have less consumer power, and often poorer knowledge of navigating the health system. AMD may coincide with other chronic diseases which appear at advanced age.

Israel has universal health coverage through its National Health Insurance (NHI) law with four health maintenance organizations (HMOs) operating as insurers and providers of services and a mix of government, public, private and health-fund owned and operated hospitals. The HMOs provide a uniform list of health services, known as the "Health Basket", for all members. A large proportion of the Israeli population have additional health insurance. Around 80% hold supplementary insurance through the HMOs, and 35% pay for private commercial health insurance; 32% have both. Globally, as more new medical technologies become available, with increasing costs, inevitably the latest and most effective treatments are not always able to be publicly funded, requiring use of complementary or private insurance to subsidize treatment.

### Aim

This study aimed to explore the perspectives of ophthalmologists working in the Israeli public healthcare system regarding timely treatment of patients with AMD.

## Methods

A qualitative research design was chosen, to allow for in-depth understanding of the issues regarding care pathways and challenges in providing timely care. The following stages were conducted:

1. Consultation with a senior ophthalmologist (HVL) and a researcher experienced in issues of access to care (RWM) about the milestones of diagnosis and care for AMD and obstacles to timely care that should be explored, respectively.
2. Design of a semi-structured interview guide including around 20 questions regarding wait times, treatment of urgent cases, the treatment process, and barriers to timely treatment.
3. Interviews were conducted between July 2020 and July 2021 with a total of 22 senior ophthalmologists. Ten participants were senior ophthalmologists or ophthalmology department heads from 10 different general hospitals across Israel’s public healthcare system and 12 were community ophthalmologists from two of the four HMOS. All interviewees specialize in retinal diseases and work with AMD patients. All geographic regions were represented (North, South, Centre/Tel Aviv, Jerusalem).

Interviews were transcribed and the transcripts carefully analysed by the authors. Details of the usual patient pathways involved in the diagnosis and treatment of AMD were obtained from participants and are described below. Emerging themes were identified according to Braun and Clarke’s approach to thematic analysis, an iterative method for identifying and reporting patterns as themes in qualitative data [9]. Themes emerged relating to barriers and obstacles to timely treatment, regional disparities, use of emergency services, the burden of accumulating cases, and issues with funding of second-line treatment. Themes were discussed and revised by all the authors.

## Results

Based on the interviews, we describe the usual referral and treatment pathway:

1. *Referral from an ophthalmologist* – The patient arrives complaining of deteriorating vision and undergoes a full eye examination by a community ophthalmologist including pupil dilation for diagnosis. Ophthalmologists sometimes - but not always - write on the referral ‘suspected (wet) AMD’, which speeds up processing and likelihood of fast treatment. In some areas, wait time for an eye doctor are long, extending the time to treatment initiation. The community ophthalmologist can either refer to optical coherence tomography (OCT), or refer directly to the retina clinic.
2. *Imaging* – OCT is a routine exam required for the diagnosis and follow-up of patients with AMD. OCT is performed either in the community or at a hospital outpatient clinic. Waiting times (WT) for OCT in the community are generally longer, so if the referral was to community OCT, it will likely delay the start of treatment. There are sometimes also delays in getting the OCT report, which requires a trained ophthalmologist.
3. *Confirmation of diagnosis by a retina specialist* – required for definite diagnosis of AMD. In some regions there is a lack of retina specialists, which causes long WT and delayed treatment. In some hospitals the wait to see a retina specialist can be 2-3 months. Therefore patients sometimes arrive via the ER as a point of entry to the hospital-based system of ophthalmic care.
4. *Treatment initiation – intraocular injections - *Patients make an appointment for treatment initiation. At the wet stage, it is important to begin treatment within a week to 10 days. The first line of treatment is Avastin, provided to the vast majority of patients. Avastin is provided without cost as part of the basket of health services covered by National Health Insurance. Lucentis, the second line treatment, is more problematic regarding co-payments, since it is very expensive (equivalent of USD 1000 per injection) for the individual patient who does not have private health insurance (commercial or supplementary health insurance, the latter offered by the HMOs). [10]
5. *Arrival via ER* – Many patients obtain treatment via the Emergency Room (ER), which often leads to faster diagnosis and initiation of injections, rather than waiting for an outpatient appointment. Sometimes the referring community ophthalmologist will refer patients to the ER as they know it is a faster route to treatment, especially with hospitals known to have longer waiting lists.

### Israel’s AMD landscape – main challenges

#### Regional disparities

Differences between hospitals and between regions were evident in the testimony of the ophthalmologists interviewed, highlighting somewhat of a "postcode lottery” (i.e., different chances of getting timely care in different geographic areas) in AMD treatment in Israel.

**Table:** 

Regional disparities – personnel	“There are huge gaps between the centre and the periphery. Patients come from the North for a consultation [to a large hospital in the central region]; there are 3 hospitals in the North, with small ophthalmology departments... patients come here after they waited months in the North”
	“There aren’t enough retina specialists, maybe 1 or 2 in the hospital in the North, there is an availability issue.” [testimony of a retina specialist in a central hospital]
Regional disparities – authorization of medication	“The process of authorization of treatment is slower in the North, it causes delays”

In the central region, and larger hospitals, treatment occurs within an acceptable time (for example, in two large hospitals in the Tel Aviv region, care was usually initiated within one or two weeks of referral), with few bottlenecks along the way. In this region, the number of ophthalmology clinics and retina experts has increased in recent years, reducing - or preventing the increase - of waiting times.

In more rural areas, there is a lower proportion of retina experts per population, both in the community and hospital settings, which leads to long delays, and eventually might result in poorer outcomes. The insufficient number of community specialists results in more patients utilizing the hospital ambulatory clinics for diagnosis and injections. Most ophthalmic specialists are not retina specialists – a sub-specialty required to make a diagnosis of AMD.

#### Waiting times

Physicians discussed the importance of starting treatment quickly for urgent cases with wet stage AMD. Many described how the most urgent cases do tend to receive timely treatment, though several factors may delay treatment.

In most of the larger, more central medical centers, WT from referral by a community-based ophthalmologist to admission in a specialty retina clinic are short, around a week to ten days. However, in some of the smaller peripheral hospitals WT can be a month to six weeks, which is considered too long. Furthermore, even in some of the larger central hospitals, a patient can wait more than a month if the referral is incomplete or inaccurate, i.e. not stating the problem as urgent or specifying “wet AMD”. Waiting time can also be delayed by the time taken to get authorization (voucher covering the costs) from the HMO for the imaging test (OCT) which is crucial for the diagnosis, or for the outpatient clinic visit. Following diagnosis and treatment initiation, delays might be caused when patients need to provide HMO authorization for the purchase of second-line medication, or need to find alternative funding if they do not have private insurance that covers most of the cost of the medication.

Some physicians refer to the retina clinic but not to the OCT imaging test, or to OCT and then to the retina clinic, instead of referring to both at the same time. This is another reason for delayed diagnosis and care. Patients with comorbidities are further at risk. Coinciding ophthalmic issues, such as diabetic retinal problems or cataract, may make diagnosis of AMD more challenging. Furthermore, some ophthalmologists refer to a general ophthalmology outpatient clinic, not to a specialty retina clinic, thus delaying diagnosis and initiation of care.

**Table:** 

Effect of delays in treatment	“A patient who waited half a year before coming to get the injection, will benefit a lot less from the treatment, because a large part of the damage to vision is irreversible.”
	“If it’s suspected to be in the wet stage they must receive the injection within a week to 10 days at most”
Barriers and bottlenecks: lack of retina specialists	“The biggest barrier is wait time until they see a retina specialist. As soon as they see the specialist they get treated quickly.”
	“The bigger problem is with examinations, not with the injections. In the morning there are specialists and interns in the retina clinic, in the afternoon there is no specialist ”
	“There is a lack of retina specialists. There are two in our hospital, one full-time and one half-time” [Northern hospital]
	“There are hardly any retina specialists in the area. Community ophthalmologists – who are mostly not retina specialists – don’t refer for injections, but refer first to OCT and to a retina specialist for diagnosis.”
Barriers and bottlenecks: OCT imaging in the community	“If they are referred to OCT, there’s a delay of about two weeks”
	“Sometimes community doctors refer to OCT imaging, it’s essential for final diagnosis. There is mediocre availability, it can be one or two months’ wait, it can take time to get the results too.”
	“There are long waits for OCT, it can be 2 months” [community ophthalmologist, Southern region]
Urgent cases receive fast treatment	“Everyone who comes with a referral for AMD gets quick treatment” [Southern hospital]

#### Effect of the COVID-19 pandemic

During the national lockdowns, some hospitals provided home visits for intra-ocular injections, and others arranged mobile units, to prevent vision lost due to discontinuation of care, but many did not provide this service. The number of patients attending for injections dropped briefly during the first wave of pandemic activity (March-April 2020), but quickly returned to normal. Some hospitals called patients who missed appointments to emphasize the importance of continuing treatment, stressing the preventive measures taken by the hospital staff to protect patients from contracting the virus. Other centers reported on clinics being closed, patients missing appointments and a disruption in continuity of care.

**Table:** 

Treatment during COVID-19 pandemic	“A minibus was donated, one doctor and one nurse did the rounds, 8 injections in patients’ homes in five hours”
	“Lots of people cancelled appointments, they didn’t get treated in time, there was a lack of continuity of treatment, clinics were closed for a whole month during the first lockdown.”

#### Copayments system: Costs and Funding of second line medicines

Interviewees discussed how in cases where a patient is not responding well to first line, they should be offered second-line treatment, which is very expensive and not covered by national health insurance. The problem of copayments for second-line medicines means not everyone gets optimal treatment. Even with complementary or commercial insurance, the patient contribution (out-of-pocket co-payment costs) for this medication is high. If patients do not have any kind of private insurance, or do not know how to navigate the system to utilize existing insurance, they may not receive the best or most appropriate treatment. The proportion of health consumers with complementary insurance varies by sociodemographics: 50% in the lower and 90% in the upper income groups; and 38% and 86% among Arab and Jewish populations, respectively [11]. In the geographic periphery, with a larger proportion of lower socio-economic status (SES) population, fewer patients have private insurance. Patients are thus more at risk of bearing the burden of uncovered medication costs, often resorting to assistance from non-governmental organizations (NGOs) that support medication expenses, or foregoing treatment altogether due to financial constraints.

**Table:** 

Issues with funding second-line treatment	“An older patient without a family member to help them with the system won’t get the form 17 [reimbursement form] and might forgo the second-line treatment.”
	“Whoever fails with first line treatment, to get the second-line, if they don’t have complementary insurance they are referred to a charity for funding, it’s very problematic.”
	“We encourage patients to take out insurance coverage. There aren’t many that don’t get second-line because of lack of insurance”

#### Inappropriate utilization of emergency services

Numerous physicians referred to the ER as point of entry for treatment. Though AMD is not considered an acute urgent condition requiring ER treatment, in some regions a high percentage of patients are referred via the ER in order to overcome delays and receive more timely treatment. One hospital in the North reported that 90% of patients with wet AMD are referred via the ER. Other hospitals reported a much lower proportion. According to the interviewees, presenting at the ER results in fast treatment initiation; if the patient arrives during the morning shift, they will receive the first injection later on the same day. From a professional and managerial perspective, this pathway is a waste of a scarce resource that might cause congestion and sub-optimal care for urgent cases.

**Table:** 

Use of the ER as entry point for treatment	“There’s a problem with the system if the majority arrive via the ER” [periphery hospital]
	“In principle it shouldn’t be through the emergency room but out of responsibility to the patient, if there’s a two month wait for the retina clinic, so they come to the ER to bypass it”
	“If it’s urgent we treat them in the ER” [about 10% of newly diagnosed patients, a central hospital]
	“Acute cases are always referred via the ER, if it’s in the morning they get the injection the same day.” [periphery hospital – reported 90% through ER for wet AMD cases]
	“If I’m sure it’s AMD I send them via the ER to do OCT, and to start treatment” [community ophthalmologist – Centre region]
	“The wait for injections isn’t too long, but it can take two months to see a retina specialist at the hospital, in the North, so it’s preferable via the ER” [community ophthalmologist – clinics in North and South periphery]

#### Accumulating cases

Each hospital receives several new AMD cases per month, but treatment is ongoing. For example, at a hospital in the South, the interviewed physician explained that there are around 10 new cases a month, equal to the number reported in two large central hospitals, and every month these join the existing cases, requiring regular injections. In contrast, in a hospital in the North, the interviewed physician reported only around 1 new patient every week or two, and a second Northern hospital reported 3-4 new patients per month, joining the hundreds already on treatment. Physicians referred to the increasing case burden - given the growing numbers of patients requiring regular injections, the total number of cases requiring treatment is continually rising. This rise is augmented by the ageing of the relatively young Israeli population, while the number of treating physicians does not rise accordingly, putting more and more pressure on existing teams. In the near future, ophthalmology departments will need more resources to deal with the ever-increasing number of AMD patients, or alternatively, less resource-intense treatments to prevent deterioration of vision in this population.

**Table:** 

Lack of trained staff/specialists	“The number of patients that need injections keeps going up – but the number of jobs doesn’t increase”
	“The number of patients goes up and up, they don’t fund more job openings accordingly, there is a limit to the response we can give”

#### Patient-related delays in seeking treatment

Physicians also mentioned patient-related delays in seeking treatment as patients often fail to notice or are unaware of the changes in their vision. AMD is sometimes diagnosed at a routine check-up.

**Table:** 

Delays in seeking treatment	“Younger people come at a later stage, because they didn’t consider it a serious problem. If there’s a family history they’re more like to come and get checked.”
	“People are not aware, they’re not sure when the deterioration in their vision started”

## Discussion

The advent of anti-VEGF treatment for preventing vision loss in patients diagnosed with AMD over the last decade has revolutionized treatment, turning this into a chronic disease which however requires ongoing treatment, for many years. New patients are added to the list every day, while existing patients remain on the list, which gets longer and longer. Furthermore, intra-ocular injections, performed by the same team, are also required for patients with diabetes, whose numbers are also on the rise. The healthcare system will need to assess future resources to cope with accumulating burden of AMD cases over time in an ageing population.

Physicians and managers in ophthalmology outpatient and hospital departments expressed the need for more trained staff (i.e. retina specialists) for the diagnosis and treatment of AMD, nationwide, and especially in the more rural locations, where a dearth of retina specialists is already evident, exacerbating disparities. Physicians additionally emphasized the importance of precise referral information, and simultaneous referral to imaging and retinal clinics, which may minimize delays in treatment.

Research in other countries has also examined barriers to optimal AMD care. A qualitative study in Australia classified barriers to effective AMD care into 3 categories – structural or health-system related factors such as costs/funding and access to services; clinician-centered factors including the referral pathway, communication issues, skills and knowledge; and patient-centered factors such as compliance, denial, trust and lifestyle changes [12]. The barriers that arose in the current study in the Israeli healthcare system were more system-based, and some clinician-based factors to be addressed, rather than patient factors.

However, awareness of AMD symptoms and the importance of early intervention could be better highlighted by a public health campaign, particularly among high-risk groups (for example smoking has been noted as an independent risk factor [5]). A UK study sought to discover the reasons for delay in diagnosis of AMD, and found that in around a third of cases, patients were unable to self-detect symptoms; and of those who did, only half sought help quickly [13]. This indicates that delays may already occur before the patient reaches the ophthalmologist. This difficulty in diagnosis may be more acute in patients with additional comorbidities.

Some limitations should be considered – participants were a small sample of senior ophthalmologists (including heads of departments) though efforts were made to recruit from different types of hospitals (governmental and HMO-owned) and from different regions (centre and periphery). Regarding community physicians, two out of the four HMOs were represented. Interview data were kept anonymous to allow participants to speak freely.

## Conclusion

Loss of vision, or decreased vision, results in dependency and reduced quality of life, as well as incurring high societal and healthcare costs. Health systems, including Israel’s public health system, will need to look ahead and increase available resources to cope with an increasing cumulative caseload for AMD in the coming years. Israel already has fewer physicians per population than the OECD average (3.19 vs 3.5 per 1000 in 2021), and certain specialties, including retina, are underpowered [14]. A better distribution of staff and resources may be necessary to address regional inequalities. HMOs may need to assess whether patients with AMD – including those without complementary insurance, frequently the weakest segments of the population – are getting the most optimal treatment, when second-line medication is required. Physicians could help their patients access more timely care, for example with more precise referral information, and simultaneous referral to imaging and retinal clinics, to avoid unnecessary delays in treatment. Clear guidelines on this may need to be issued by professional associations.

With regard to patients, it may be necessary to design a campaign to raise awareness of the importance of consulting an ophthalmologist for decreasing visual acuity, often perceived as a “normal ageing symptom”. Patients should be empowered to recognize and acknowledge typical symptoms of AMD and act accordingly, particularly in high-risk groups.

The major policy implications of the current study relate to the urgent need of the healthcare system to assess available and future resources to cope with increasing demand in the retina specialty in an ageing population. This includes the consideration of ways to increase the number of physicians choosing this specialty, for example incentivizing neglected specialties, as well as horizon scanning for new technologies in the field, for more effective and less resource intense treatment for AMD.

## Data Availability

The datasets analysed during the current study available from the corresponding author on reasonable request.

## Abbreviations

AMD: Age-related macular degeneration
ER: Emergency room
HMO: Health maintenance organization
NHI: National Health Insurance
OCT: Ocular coherence tomography
VEGF: Vascular endothelial growth factor
